# Radiomics Analysis of Postoperative Epilepsy Seizures in Low-Grade Gliomas Using Preoperative MR Images

**DOI:** 10.3389/fonc.2020.01096

**Published:** 2020-07-08

**Authors:** Kai Sun, Zhenyu Liu, Yiming Li, Lei Wang, Zhenchao Tang, Shuo Wang, Xuezhi Zhou, Lizhi Shao, Caixia Sun, Xing Liu, Tao Jiang, Yinyan Wang, Jie Tian

**Affiliations:** ^1^Engineering Research Center of Molecular and Neuro Imaging of Ministry of Education, School of Life Science and Technology, Xidian University, Xi'an, China; ^2^CAS Key Laboratory of Molecular Imaging, Institute of Automation, Beijing, China; ^3^Beijing Tiantan Hospital, Capital Medical University, Beijing, China; ^4^Beijing Advanced Innovation Center for Big Data-Based Precision Medicine, School of Medicine, Beihang University, Beijing, China; ^5^School of Computer Science and Engineering, Southeast University, Nanjing, China; ^6^Key Laboratory of Intelligent Medical Image Analysis and Precise Diagnosis of Guizhou Province, School of Computer Science and Technology, Guizhou University, Guiyang, China; ^7^University of Chinese Academy of Science, Beijing, China

**Keywords:** low-grade glioma, epilepsy, radiomics, elastic net, Cox regression

## Abstract

**Purpose:** The present study aimed to evaluate the performance of radiomics features in the preoperative prediction of epileptic seizure following surgery in patients with LGG.

**Methods:** This retrospective study collected 130 patients with LGG. Radiomics features were extracted from the T2-weighted MR images obtained before surgery. Multivariable Cox-regression with two nested leave-one-out cross validation (LOOCV) loops was applied to predict the prognosis, and elastic net was used in each LOOCV loop to select the predictive features. Logistic models were then built with the selected features to predict epileptic seizures at two time points. Student's *t*-tests were then used to compare the logistic model predicted probabilities of developing epilepsy in the epilepsy and non-epilepsy groups. The *t*-test was used to identify features that differentiated patients with early-onset epilepsy from their late-onset counterparts.

**Results:** Seventeen features were selected with the two nested LOOCV loops. The index of concordance (C-index) of the Cox model was 0.683, and the logistic model predicted probabilities of seizure were significantly different between the epilepsy and non-epilepsy groups at each time point. Moreover, one feature was found to be significantly different between the patients with early- or late-onset epilepsy.

**Conclusion:** A total of 17 radiomics features were correlated with postoperative epileptic seizures in patients with LGG and one feature was a significant predictor of the time of epilepsy onset.

## Introduction

Low-grade gliomas (LGGs) are slow-growing, infiltrative tumors frequently associated with seizures. Up to 90% of the patients with LGG may have seizures as the initial presentation leading to tumor diagnosis. Additionally, epilepsy seizure could be used as a clinical predictor for early tumor recurrence with LGGs (1). The persistence of seizures in these patients may be associated with the worsening of their neurological, neuropsychological, and psychological status, reducing their quality of life (2, 3). With a prolonged duration of epilepsy, the symptoms of LGG may spread to the neocortex and subcortical structures, gradually disrupting normal brain networks and causing dysfunction of both the peritumoral area and remote brain tissues (1). The reported recurrence of epilepsy in 20–40% of patients demonstrates that the current conventional treatment strategy used to address glioma-related epilepsy, including antiepileptic drugs (AEDs) and anti-tumor therapies, remain unsatisfactory (4–6). The application of AEDs has been a controversial issue for a long time, there still has no strategy for individualized prophylactic. Thus, the preoperative prediction of epilepsy seizures following surgery could help clinicians evaluate the risk of patients for epilepsy after surgery, further to make decision of individualized treatment strategy, which was significant for clinical treatment.

Several studies identified risk factors for the development of gliomas related epilepsy: tumor location, tumor histology, microenvironment, and genetic mutation (4, 7–9). Some investigations have used medical imaging to study the correlation between LGG and epilepsy (10, 11), and Wang et al. developed a probabilistic risk atlas of gliomas related epilepsy (8). However, few studies evaluated the correlation between these factors and the risk of epilepsy seizure after surgery. In addition, research has neglected temporal concerning the condition—despite the value of such information to enhance the timeliness of interventions.

The development of pattern recognition technology has driven the development of medical imaging data analysis, and advances in data mining and machine learning have rendered it possible to convert medical images into minable data. The concept of “radiomics” first described in 2012 (12, 13), calls a comprehensive analysis of medical images (14). By converting medical images into high-dimensional, mineable, and quantitative imaging features, radiomics could offer large amounts of information inaccessible to the human eye. Recently, radiomics had been widely and successfully applied to inform decision making in the treatment of tumors and neuropsychiatric diseases (15–17), including in gliomas and epilepsy (18, 19). Liu et al. built a radiomics signature using quantitative imaging features to predict LGG-related epilepsy (20). Based on the successful application, we hypothesized that radiomics analysis could provide a chance for preoperative prediction of epilepsy seizures following surgery in LGG patients. Hence, by incorporating the temporal information, the model could provide a timelier and finer prediction for the intervention.

In this study, we retrospectively collected pretreatment neuroimaging data and prognosis outcomes of 130 patients with LGG who underwent surgery. We attempted to explore the relationship between radiomics features and the prognosis of postoperative epilepsy by performing a multivariable analysis.

## Methods

### Patients

A total of 130 patients who were surgically treated at Beijing Tiantan Hospital between October 2005 and August 2008 were collected in this retrospective study. The tumor types of all patients were identified according to WHO 2016 classification. The inclusion criteria were set as: all patients underwent standard surgery and were diagnosed with LGGs confirmed by postsurgical histopathology. To ensure that prognoses were accurate for each patient, we assured that at least two years of follow-up records were available for each patient. All patients were postoperatively followed up every 6 months during the first year, and then annually thereafter. The Ethics Committee of the Beijing Tiantan Hospital approved the study, and written informed consent was obtained from all participants.

### Brain Imaging and Tumor Masking

The feature extraction in the current study followed the Image Biomarker Standardization Initiative (IBSI) guideline (21). T2-weighted images were obtained on a Magnetom Trio 3.0T scanner (Siemens, Erlangen, Germany) with a 12-channel receive-only head coil. The parameters were set as follows: repetition time = 5,800 ms; echo time = 110 ms; flip angle = 150 degrees; 24 slices; field of view = 240 × 188 mm^2^; voxel size = 0.6 × 0.6 × 5.0 mm^3^; matrix = 384 × 300. Tumors were semi-automatically segmented along the lesion contour on each patient's T2-weighted images in native space by at least two experienced neuroradiologists using the ITK-SNAP software (v 3.6.0; www.itksnap.org), while two other board-certified experts reviewed the segmentations using imaging features in combination with seizure history, clinical examination, neuroimaging data to solve any discrepancies. The areas with abnormal hyperintense signals on the images were identified as tumor volumes, and the cerebrospinal fluid signals should not be involved in. When the concordance between the tumor masks of one patient identified by the two neuroradiologists was higher than 95%, the tumor masks were combined.

### Feature Extraction

The delineated tumor area was used as the region of interest (ROI) to extract radiomics features. The features were calculated with the largest slice of ROI. A total of 4,650 features were extracted. These features could be divided into 4 types: shape-based features, first-order statistical features, textural features and wavelet features. Detailed information and formula are described in the supplemental content. The radiomics feature extraction was performed using in-house software written in MATLAB 2017b (MathWorks, Inc., Natick, MA, USA).

### Feature Selection and Model Development

This study aimed to find the discriminative radiomics features capable of predicting epileptic seizures after tumor resection, and further to distinguish early-onset seizures (patients who would develop epilepsy within 6 months after surgery) from late-onset ones (patients who would develop epilepsy more than 6 months after surgery). In order to take full advantage of the temporal information, this study adopted a proportional hazards model (Cox model) (22). The end point of this study was disease-free survival (DFS), which was defined as the time from the date of surgery until the date of epileptic seizures (event) or until the latest date at which the patient was known to be free of epilepsy (censored).

Since the dimensionality of the radiomics feature space was high, and could easily lead to over-fitting or bias in the multivariable analysis, dimensional reduction was necessary to ensure the reliability of our results. All features were initially normalized as z-scores. After normalization, we performed univariate Cox regression on each feature, retained the significant features (*P* < 0.05). Then elastic net (E-net) Cox regression was used as a multivariable analysis to choose the most important features. The E-net penalty was a weighted sum of the least absolute shrinkage and selection operator (LASSO) penalty and ridge penalty, which retained the advantage of feature selection while at the same time, compared with LASSO, avoiding the interference of feature collinearity. The relative weight of LASSO and ridge penalty was selected to maximize the Cox's log-partial likelihood (23):

$$\begin{array}{l} \sum_{i=1}^{n}\delta_{i}\left\{{X^{\prime }}_{i}\beta -log\left[\sum_{j\in R\left(t_{i}\right)}\exp\left({X^{\prime }}_{j}\beta \right)\right]\right\}-\lambda [\alpha {\left\|\beta \right\|}_{1}\\ \;+0.5\left(1-\alpha \right){\left\|\beta \right\|}_{2}^{2}] \end{array}$$

where *t*_*i*_ was the survival time (observed or censored) for the *i*th patient, *R*(*t*_*i*_) was the risk set at time *t*_*i*_, $X_{i}={\left(X_{i1},\cdots \;,X_{ip}\right)}^{\prime }$ was the regression vector of p-variables for the *i*th patient, $\beta ={\left(\beta_{1},\cdots \;,\beta_{p}\right)}^{\prime }$ was the column vector of the regression parameters.

**Figure 1:**
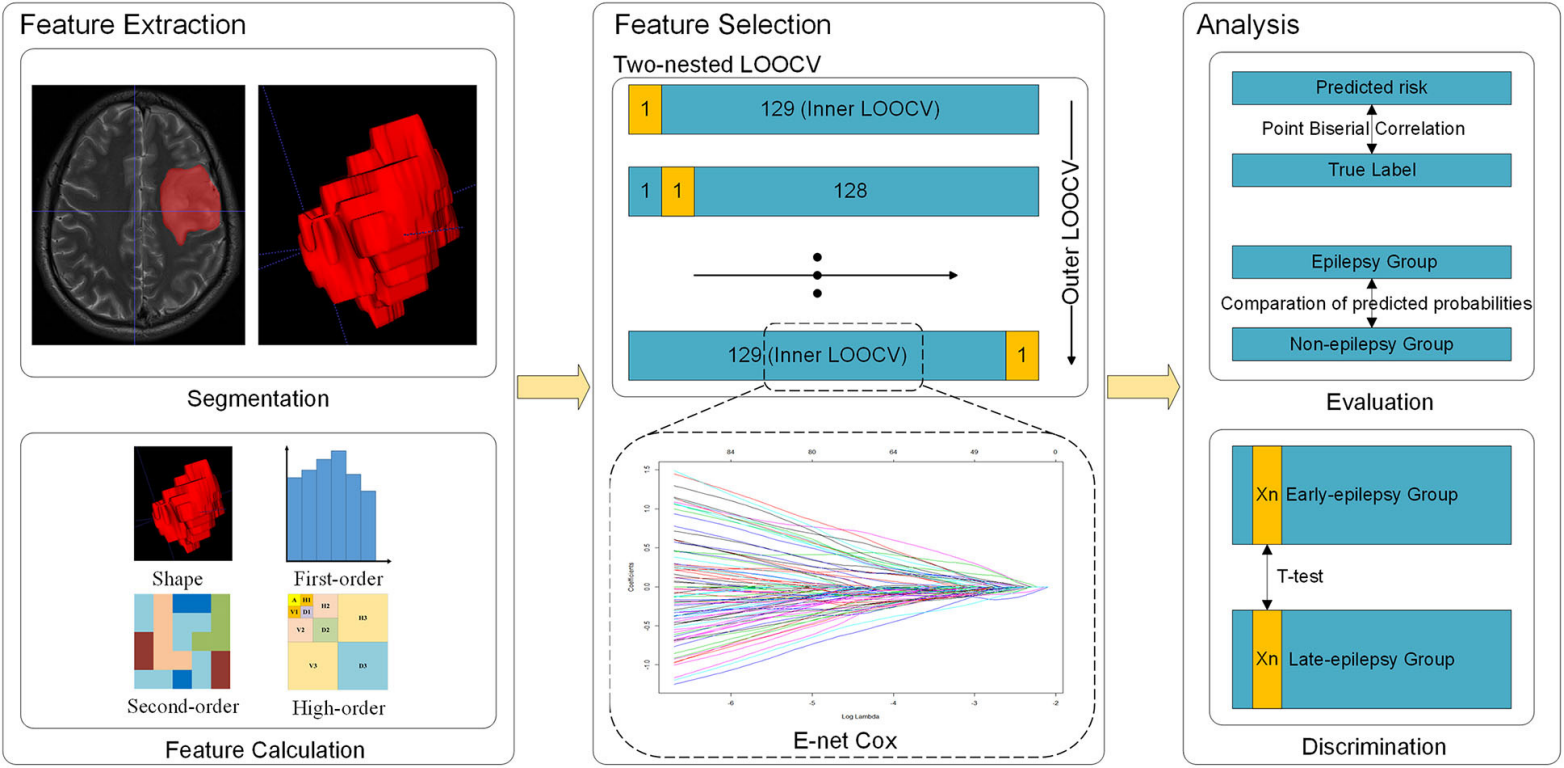
Flow chart of the study.

Since the sample size of this study was relatively small, we used two nested leave-one-out cross validation (LOOCV) to maximize the utilization of samples and to select the regularization parameters (Figure 1) (24, 25). In the outer LOOCV loop, each subject in turn was left out as the validation set, the other 129 being used to train an optimal E-net Cox model using the inner LOOCV loop. Specifically, the inner LOOCV loop was performed to train the optimal E-net Cox model with a pair of best parameters λ and α maximizing the Cox's log-partial likelihood based on the training data (all 130 samples excepting the one reserved for validation). We thus obtained 130 different models, since the training samples used in each model training were not identical. The features retained in more LOOCV loops were more stable, implying that they played more important roles. Therefore, we chose the features which were retained in at least 90% of the loops (117 loops) as the most important ones.

The features thus selected were used to build a Cox model and the Harrell concordance index (C-index) was calculated to evaluate its performance (26). We also built two separate logistic models to predict the status at 6 and 24 months using LOOCV, since the 6-month time point was used to define early-onset and the 24-month time point represented the longest available follow-up time for the whole dataset. We then computed the point-biserial-correlation between the probabilities of epilepsy predicted by the logistic model and the true labels (27), computing the correlation coefficient and the corresponding *P*-values. Additionally, we divided the patients into epilepsy and non-epilepsy groups based on the true label, and used the Student's *t*-test to compare the predicted probabilities between the two groups separately at 6 and 24 months. Finally, Student's *t*-test was performed on each selected feature to identify differences between the patients with early- or late-onset of epilepsy.

To evaluate the impact of preoperative epilepsy on radiomics features, we performed a subgroup analysis based on preoperative seizures status. Specifically, we used Student's *t*-test to compare the predicted risk of the Cox model between preoperative epilepsy and no-epilepsy groups.

Since the pathological type, tumor location and the tumor volume could be the risk factors that may lead to postoperative epileptic seizure, we further investigated the correlation between the selected radiomics features with clinical factors.

## Results

### Demographic and Clinical Data

According to the follow-up records, 51 patients were considered to have developed epilepsy by the end of the follow-up, specifically, 41 patients within 6 months after surgery, 10 patients within 6 to 24 months after surgery, and 79 patients were seizure-free through the end of the follow-up period. We collected six main clinical factors, age, sex, tumor location, the status of preoperative epilepsy, the tumor pathology types and the IDH status. There was no significant difference in the clinical factors between the epileptic and non-epileptic groups (Table 1). The age range of the patients was from 18 to 68 when they did the surgery.

**Table 1:** Demographics and clinical characteristics of patients with low-grade gliomas.

	**Epilepsy recurrence**	**Non-epilepsy recurrence**	***P***
**Age (mean** **±** **deviation)**	37.667 ± 9.651	38.101 ± 9.517	0.802
**Sex**			0.856
Male	30	49	
Female	21	30	
**Tumor location**			0.365
Frontal lobe	36	60	
Temporal lobe	23	22	
Parietal lobe	4	9	
Insula	8	9	
**Preoperative epilepsy**			0.342
Yes	39	53	
No	12	26	
**Tumor pathology**			0.725
*Diffuse Astrocytoma*			
IDH-mutant with 1p/19q no-deleted	17	31	
IDH-wildtype	10	13	
*Oligodendroglioma*			
IDH-mutant with 1p/19q co-deleted	16	27	
*NOS*	8	8	
**IDH status**			0.875
IDH-mutant	41	64	
IDH-wildtype	10	13	

*The status of epilepsy recurrence is determined at 24 months. The location of the tumor is determined by the region of the tumor involved, where some patients have tumors involving multiple regions. Chi-Square test or Student's t-test were used to compare the differences between epilepsy and non-epilepsy groups*.

### Feature Selected

The features were selected by E-net cox in each inner LOOCV loop, and the loop in which each feature was retained was recorded. After feature selection, we retained 17 features. The number of LOOCV loops in which each feature were selected is listed in Table 2. The features selected by more loops were considered more robust.

**Table 2:** The features selected by the two-nest LOOCV loop.

**Name**	**The number of loops**	***P***
gabor3_glszm_SZHGE	117	0.007^*^
gabor7_glcm_cluster_shade	123	0.247
gabor14_glcm_cluster_tendency	129	0.461
gabor18_glcm_IMC2	122	0.591
gabor18_glszm_LZLGE	120	0.564
gabor29_glszm_SZSE	129	0.406
gabor29_glszm_SZHGE	125	0.375
gabor30_glszm_SZHGE	117	0.692
gabor35_glszm_SZLGE	129	0.510
gabor36_glrlm_LGLRE	119	0.979
gabor36_glrlm45_LGLRE	121	0.958
gabor36_glszm_LGLZE	129	0.402
W5S5_fos_skewness	123	0.486
W5S5_fos_mass	129	0.919
W5W5_fos_mean	128	0.431
W5W5_fos_mass	129	0.853
R5S5_fos_median	129	0.847

*The number of loops indicates how many LOOCV loops have selected the feature. The P-value is calculated by the Student's T-test between the early- and late-onset of epilepsy patients*.

* *Significant difference*.

### Performance

**Figure 2:**
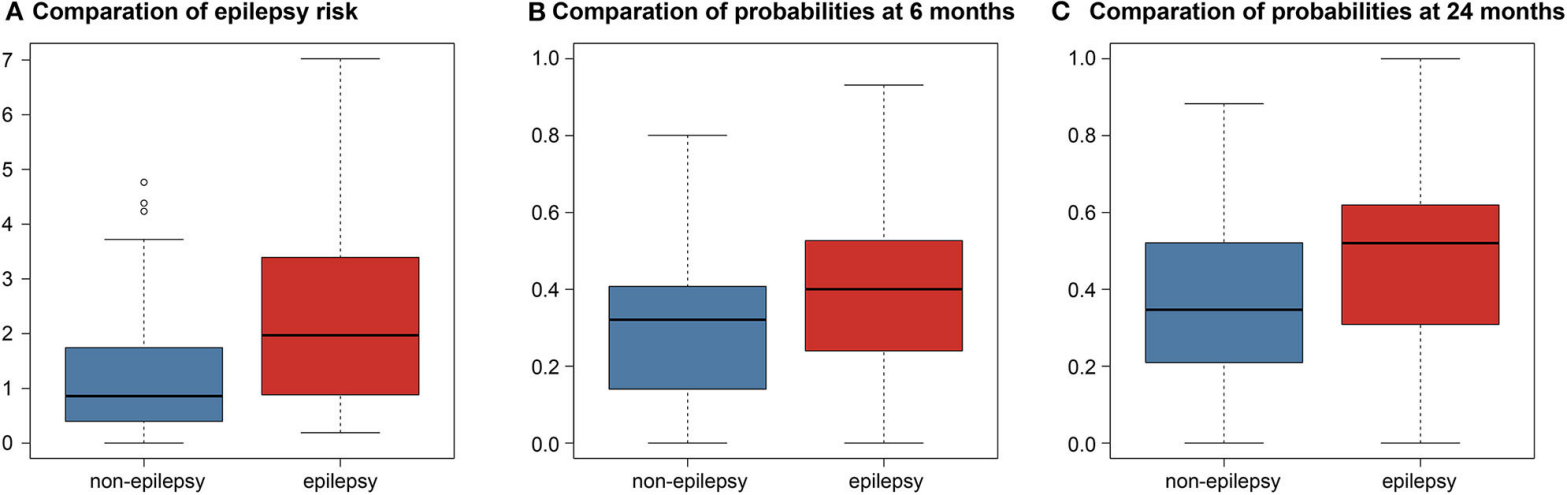
Boxplots comparison differences between predictions in the non-epilepsy and epilepsy groups. **(A)** Comparation of epilepsy risk. Boxplot of the predicted risk values of the Cox model corresponding to both the no epilepsy and epilepsy groups. **(B)** Comparation of probabilities at 6 months. Boxplot of the predicted probabilities of the logistic model at 6 months. **(C)** Comparation of probabilities at 24 months. Boxplot of the predicted probabilities of the logistic model at 24 months.

The C-index of the Cox model built with the features selected by the two-nested LOOCV was 0.683, meaning that the selected features were predictive for the risk of epileptic seizures after surgery in LGG patients. When the predicted risk was compared between the two groups (no epilepsy and epilepsy) based on the true labels (Figure 2), the *t*-test revealed a significant difference between the two groups (*P* < 0.001).

The point-biserial-correlation between the probabilities predicted by the logistic model and the true labels was computed separately at the two time points of 6 and 24 months after surgery. At 6 months, the R- and *P*-values were 0.235 and 0.007, respectively, indicating that the selected features could predict whether patients would develop epilepsy at 6 months after surgery. Similarly, the R- and *P*-values at 24 months were 0.300 and < 0.001, respectively, indicating that the predictive power of the selected features was retained at 24 months after surgery. Moreover, the Student's t-test comparing the probabilities predicted by the logistic model between the epilepsy and non-epilepsy groups, separately at the two time points, gave *P*-values both smaller than 0.001 indicating that the features could distinguish patients with epilepsy from those without epilepsy independently of the time point (Figure 2).

**Figure 3:**
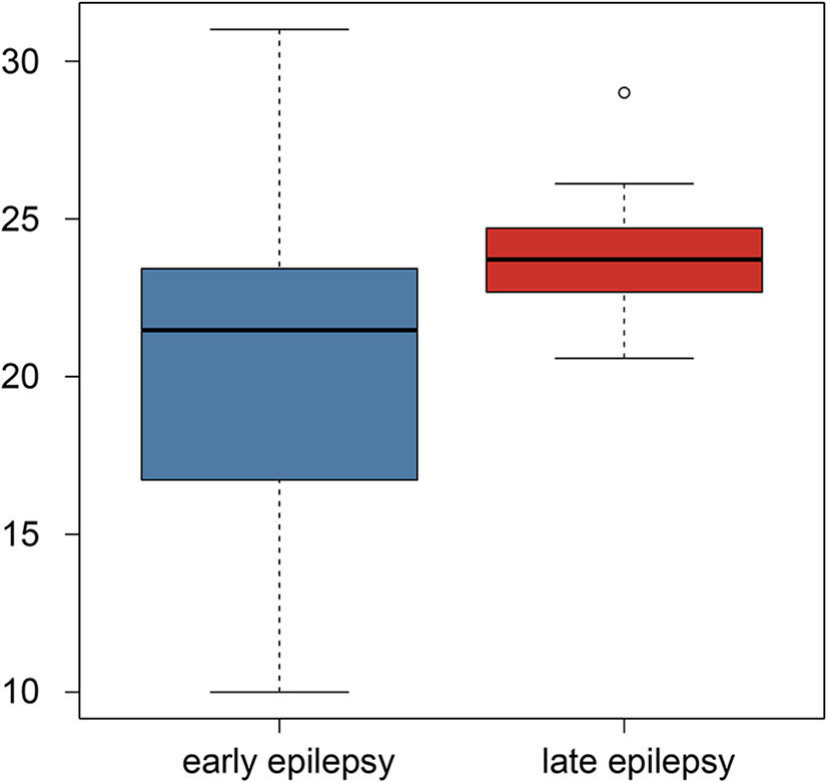
Boxplot of the selected feature comparison between the early-onset and late-onset epilepsy patients.

Using Student's *t*-test, we also compared the early- and late-onset epilepsy patients in terms of each selected feature. The *P*-values are listed in Table 2. The feature named “gabor3_glszm_SZHGE” was significantly different between the early- and late-onset of epilepsy groups, implying that the early-or late-onset of epilepsy in LGG patients could be predicted based on this feature, as shown in Figure 3 and Figure S1, the values of the early-onset group were lower than the late-onset group, indicating that the lower the value of this feature, the sooner that post-operative seizures would occur.

**Figure 4:**
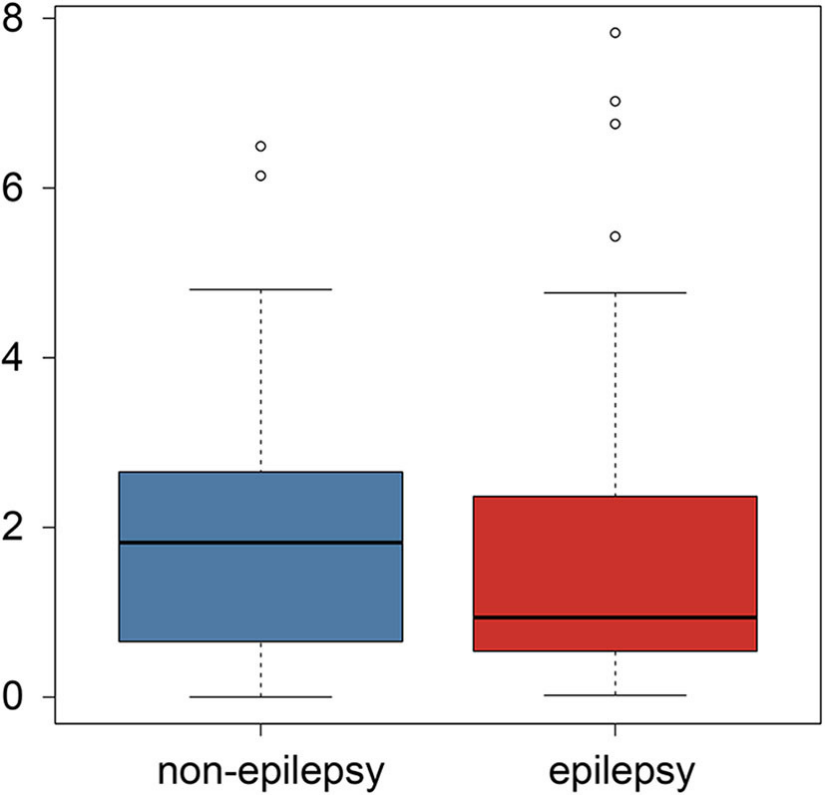
Boxplot of the postoperative epilepsy risk comparison between the preoperative epilepsy and non-epilepsy patients.

The subgroup analysis of the preoperative seizures status demonstrated that preoperative epilepsy was not associated with the predictive value of the radiomics analysis (*P* = 0.847), as shown in Figure 4.

### Analysis of Clinical Correlation Analysis

With regard to the pathological type, the corresponding R-values and *P*-values were listed in the Table S1. We found there were no correlation between the selected radiomics features and the pathological type.

To the tumor location, we determined the location of tumors based on the region of the tumors involve, and segmented into frontal lobe, temporal lobe, parietal lobe and Insula. The Students' *T*-tests were used to determine that if there were any differences in the selected radiomics features when a certain brain region was involved in or not. The results were listed in Table S2. We found that the radiomics feature named “gabor3_glszm_SZHGE” was significantly different when the tumor region located in the frontal lobe or not (*P* = 0.022).

To the tumor volume, we used the Pearson's correlation to explore the relationship between the selected radiomics features and the tumor volume. The corresponding R-value and *P*-values were listed in the Table S3. We found six radiomics features were still significant after multiple correlation. For the radiomics feature named “gabor3_glszm_SZHGE,” which was a significant predictor of early or late epilepsy onset, was also significant after Bonferroni correlation (*P* < 0.001). The result showed that this feature was negatively related to the tumor volume.

## Discussion

In the present study, we aimed to evaluate the performance of the radiomics features in the pretreatment prediction of epileptic seizure after surgery in patients with LGG. We used a two-nested LOOCV loop and recorded the frequency of the features to select the most robust and distinguishable features, obtaining in the end 17 radiomics features. The results suggested that the radiomics features could be successfully used for the pretreatment prediction of epileptic seizures following surgery. In addition, we further analyzed the correlation between radiomics features and the time of seizure utilizing the time information, and the results demonstrated that the radiomics features could be used to predict the patients with early- or late-onset epilepsy. The subgroup analysis proved that the radiomics features were not influenced by the status of preoperative epilepsy. The study details according to IBSI was reported in Table S4.

Since the introduction of radiomics, it has gained wide application to the treatment of brain tumors: from diagnosis, through treatment evaluation, to prognosis (28–30). This increased application of radiomics is attributable to its use in helping clinicians to extract more high-throughput information from medical images with higher efficacy, thus improving decision making (31). The use of radiomics has been recently extended to neuropsychiatric diseases with success, suggesting that radiomics might be employable in preoperative prediction of epileptic seizures following surgery in LGG patients. The present study extracted 4650 radiomics features which contained much high-throughput information that beyond of human eyes. By combining the high-throughput information and the time information, we utilized a Cox model to evaluate the performance of radiomics features for preoperative prediction of epileptic seizure after surgery, and the results demonstrated that radiomics could successfully be applied to the pretreatment prediction of postoperative epileptic seizures in LGG patients.

Although the radiomics features contained much information, there were also some redundant information. Hence, we used the E-net regularization to choose the features most predictive of epilepsy status following surgery. Considering the small sample size and the randomness of sample, we performed two-nested LOOCV loops. The features chosen by E-net in each loop would not be identical since the samples were not identical, thus we record the number of loops in which each of the selected features presented because they were chosen by more loops would be more robust. The 17 radiomics features were the most predictive and robust features for the pretreatment prediction of epileptic seizure following surgery, which meant these features had universality for clinical application. In addition, our findings suggest that the radiomics feature named “gabor3_glszm_SZHGE” might be the most important indicator for clinical application since it was predictive of whether and when epilepsy occurs following the operation. Additionally, the mean value of the feature was higher when the frontal lobe was involved in (25.7 in the frontal lobe involved group > 20.8 in the frontal lobe no involved group) according to the clinical correlation analysis. It implied that the larger the feature (gabor3_glszm_SZHGE) value, the later the onset time may be combining with Figure 3, which means that when tumors involved in the frontal lobe, seizure may occur early after surgery. This finding might be helpful for postoperative epilepsy prevention. The result from Table S3 showed that this feature was negatively related to the tumor volume. It implied that the smaller the feature (gabor3_glszm_SZHGE) value, the earlier the onset time may be, which means that the larger the tumor volume, the earlier the onset time based on the current analysis.

The administration of AEDs was a major prophylactic strategy used to address postoperative epilepsy. Details concerning the administration of AEDs to glioma patients, including the drug dosage and duration of prescription, depended on clinical risk factors such as preoperative GRE and tumor resection, etc. (6, 32–34). The standard of AEDs was still controversial and too broad for subjects, and lack of an accurate strategy for individuals now. The present study would help to inform research on the development of individualized prophylactic strategies. Moreover, future clinical applications of our research would help clinicians plan strategies to address the potential onset of epilepsy when treating patients with LGG: the dosages of AEDs need to be raised, and the duration prolonged. Furthermore, our methodology would allow oncologists to perform more frequent and timely follow-up observations of the patients with low values of “gabor3_glszm_SZHGE” and administer an appropriate early intervention.

This study has some limitations. First, the epilepsy status of LGG patients was diagnosed based on clinical presentation, and the patients underwent surgery shortly following diagnosis, so that many of them could not undergo electroencephalography necessary to confirm the diagnosis prior to surgery. Second, the sample size of this study is small, it is difficult to divide an independent validation dataset in this study. For this reason, we adopted a two-nested LOOCV loops to maximize the utility of our sample size and improve the credibility of this study. Despite the above limitations, the features selected in this study performed well, as shown by the C-index. We intend to use a deep learning model and a larger sample size in future studies.

## Conclusions

In conclusion, we selected 17 radiomics features that correlated with postoperative epileptic seizure in patients with LGGs, and found one feature to be a significant predictor of early or late epilepsy onset. Our findings indicate that the features we chose are useful in the management of postoperative epilepsy and that radiomics analysis can potentially be applied to the individualization of prophylactic treatment strategies that address postsurgical epileptic.

## Data Availability Statement

The datasets presented in this article are not readily available because they belong to Beijing Tiantan Hospital. Requests to access the datasets should be directed to Yinyan Wang (tiantanyinyan@126.com).

## Ethics Statement

The studies involving human participants were reviewed and approved by The Ethics Committee of the Beijing Tiantan Hospital. Written informed consent to participate in this study was provided by the participants' legal guardian/next of kin.

## Author Contributions

Study conception and design: JT and YW. Acquisition of data: YL, LW, XL, TJ, and YW. Analysis and interpretation of data: KS, ZL, ZT, SW, XZ, LS, and CS. Drafting of manuscript: KS. Critical revision: KS, ZL, YL, YW, and JT. All authors contributed to the article and approved the submitted version.

## Conflict of Interest

The authors declare that the research was conducted in the absence of any commercial or financial relationships that could be construed as a potential conflict of interest.

## Supplementary Material

Figure S1 T2-weighted images with tumor masks for two cases with similar tumor location and size. Case 1 corresponds to a patient who developed epilepsy within 6 months of surgery, the radiomics feature named gabor3_glszm_SZHGE equaled 21.8373. Case 2 corresponds to a patient who developed epilepsy within 6–12 months of surgery, the radiomics feature named gabor3_glszm_SZHGE equaled 27.0216. The non-overlaid and ROI-overlaid source images of case 1 were shown in **(A,C)** separately. The non-overlaid and ROI-overlaid source images of case 2 were shown in **(B,D)** separately.
