# Supplementary figures and images for: Radiomics Analysis of Postoperative Epilepsy Seizures in Low-Grade Gliomas Using Preoperative MR Images
Source: Front Oncol. 2020 Jul 8;10:1096. doi: 10.3389/fonc.2020.01096 (PMC7360821; doi:10.3389/fonc.2020.01096)

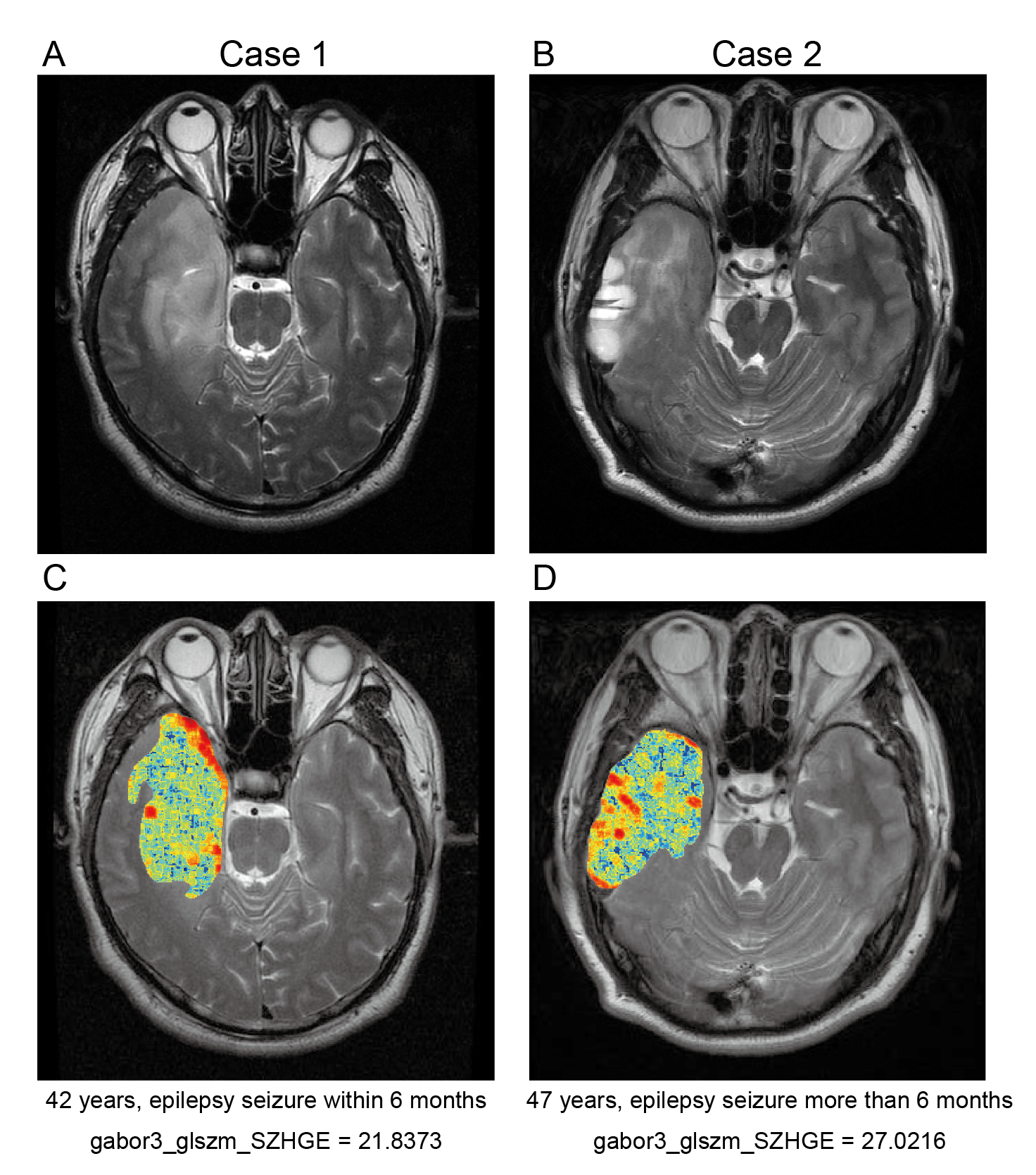

Supplement: Figure S1 — T2-weighted images with tumor masks for two cases with similar tumor location and size. Case 1 corresponds to a patient who developed epilepsy within 6 months of surgery, the radiomics feature named gabor3_glszm_SZHGE equaled 21.8373. Case 2 corresponds to a patient who developed epilepsy within 6–12 months of surgery, the radiomics feature named gabor3_glszm_SZHGE equaled 27.0216. The non-overlaid and ROI-overlaid source images of case 1 were shown in (A,C) separately. The non-overlaid and ROI-overlaid source images of case 2 were shown in (B,D) separately. [file Image_1.TIF]
