# Supplementary material for: Radiomics Analysis of Postoperative Epilepsy Seizures in Low-Grade Gliomas Using Preoperative MR Images
Source: Front Oncol. 2020 Jul 8;10:1096. doi: 10.3389/fonc.2020.01096 (PMC7360821; doi:10.3389/fonc.2020.01096)
Supplement: Supplementary file 2 [file Data_Sheet_1.docx]

**Supplementary to**

**Radiomics analysis of postoperative epilepsy seizures in low-grade gliomas based on multiple time points**

**A1. Radiomic Feature Extraction**

A total of 4650 quantitative imaging features including 8 shape-based features, 17 first-order statistical features, 90 textural features and 4535 wavelet features (4280 features of Gabor-bank wavelet filtered images and 155 features of Law’s filtered images), were extracted for T2-weighted images corresponding ROIs.

**(1) Shape-based features**

In this group of features, we included descriptors of the three-dimensional shape and size of the tumor region. Let in the following definitions V denote the volume and A the surface area of the volume of interest. We determined the following shape and size based features:

1. **Compactness 1**=$\frac{V}{\sqrt{\pi}A^{\frac{2}{3}}}$
2. **Compactness 2**=$36\pi\frac{V^{2}}{A^{3}}$
3. **Maximum 3d diameter**: The maximum three-dimensional tumor diameter is measured as the largest pairwise Euclidean distance, between voxels on the surface of the tumor volume.
4. **Spherical disproportion** = $\frac{A}{4\pi R^{2}}$
5. **Sphericity** =$\frac{\pi^{\frac{1}{3}}(6V)^{\frac{2}{3}}}{A}$
6. **Surface area**: The surface area is calculated by triangulation (i.e. dividing the surface into connected triangles) and is defined as:

$$A\text{=}\sum_{\text{i}=1}^{N} \frac{1}{2}\left|a_{i}b_{i}\times a_{i}c_{i}\right|$$

Where N is the total number of triangles covering the surface and a, b and c are edge vectors of the triangles.

1. **Surface to volume ratio** =$\frac{A}{V}$
2. **Volume**: The volume (V) of the tumor is determined by counting the number of pixels in the tumor region and multiplying this value by the voxel size.

**(2) First order statistical features**

The following 17 statistical features were extracted.

Let **X** be the three dimensional image matrix with *N* voxels of the ROI and P be the first order histogram distribution with *N_g_* discrete intensity levels.

1. **IntensityMax:** The maximum intensity value of **X.**
2. **IntensityMin:** The minimum intensity value of **X**.
3. **Median:** The median intensity value of **X**.
4. **IntensityStd:**

$$IntensityStd=(\frac{1}{L*W*H-1}\sum_{i=1}^{L} \sum_{j=1}^{W} \sum_{k=1}^{H} (X(\mathrm{i},\mathrm{j},\mathrm{k})-IntensityAve)^{2})^{1/2}$$

1. **Mean:**

$$\frac{1}{N}\sum_{i}^{N} X(i)$$

1. **Variance:**

$$\sqrt{\sum_{j=1}^{N_{g}} P(\mathrm{j})*(\mathrm{j}-\sum_{i=1}^{N_{g}} P(\mathrm{i})*\mathrm{i})^{2}}$$

1. **Skewness:**

$$\sqrt{\sum_{j=1}^{N_{g}} P(\mathrm{j})*(\mathrm{j}-\sum_{i=1}^{N_{g}} P(\mathrm{i})*\mathrm{i})^{3}}$$

1. **Kurtosis:**

$$\sqrt{\sum_{j=1}^{N_{g}} P(\mathrm{j})*(\mathrm{j}-\sum_{i=1}^{N_{g}} P(\mathrm{i})*\mathrm{i})^{4}}$$

1. **Range:**

The range of intensity values of **X**.

1. **Mean absolute deviation:**

The mean of the absolute deviations of all voxel intensities around the mean intensity value

1. **Energy:**

$$\sum_{i}^{N} X(i)^{2}$$

1. **Entropy:**

$$\sum_{i=1}^{N_{g}} P(i)\log_{2}P(i)$$

1. **Entropy_p:**

$$\sum_{i=1}^{N_{g}} \frac{P(i)}{N}\log_{2}\frac{P(i)}{N}$$

1. **Root mean square:**

$$\sqrt{\frac{\sum_{i}^{N}X(i)^{2}}{N}}$$

1. **Uniformity:**

$$\sum_{i=1}^{N_{g}} P(i)^{2}$$

1. **Uniformity_p:**

$$\sum_{i=1}^{N_{g}} (\frac{P(i)}{N})^{2}$$

1. **Mass:**

The sum intensity value of $X$.

**(3) Textural features**

Second order statistic texture features, and higher order statistic texture features were extracted. Forty-four second order statistic texture features could be calculated from the Gray Level Co-occurrence Matrix (GLCM). Forty-six high order statistic texture features were calculated from the Gray Level Size Zone Matrix (GLSZM), Gray Level Run Length Matrix (GLRLM), and Neighborhood Gray Tone Difference Matrix (NGTDM). All of the GLCM, GLSZM, GLRLM, and NGTDM based texture feature were calculated using a 2D analysis and then averaged for all slices within the three-dimensional tumor volume.

*Gray-Level Co-Occurrence Matrix based features (GLCM)*

GLCM based features were second-order statistical texture features, which are defined as a matrix *M* (*i, j; δ, θ*) to indicate the relative frequency with intensity values of pixels (*i* and *j*) at the distance of *δ* in direction *θ*.

Let:

*M*(*i, j*) be the co-occurrence matrix for an arbitrary *δ* and *θ*, set *δ=1 and θ=0 and 45*

*N_g_* be the number of discrete intensity levels in the images, set as 25,

*μ* be the mean of *M*(*i, j*),

$m_{x}(\mathrm{i})=\sum_{j=1}^{N_{g}} M(\mathrm{i},\mathrm{j})$ be the marginal row probabilities,

$m_{y}(\mathrm{i})=\sum_{i=1}^{N_{g}} M(\mathrm{i},\mathrm{j})$ be the marginal column probabilities, and *u_y ,_μ_x,_* be the mean of *m_x_* .and *m_y_*

$HX=-\sum_{i=1}^{N_{g}} m_{x}(i)log(m_{x}(i)$,

$HY=-\sum_{i=1}^{N_{g}} m_{y}(i)log(m_{y}(i)$,

$HXY=-\sum_{i=1}^{N_{g}} \sum_{j=1}^{N_{g}} m(i,j)log(m(i,j))$,

$HXY1=-\sum_{i=1}^{N_{g}} \sum_{j=1}^{N_{g}} m(i,j)log(m_{x}(i)m_{y}(j))$.

$HXY2=-\sum_{i=1}^{N_{g}} \sum_{j=1}^{N_{g}} m_{x}(i)m_{y}(j)log(m_{x}(i)m_{y}(j))$.

1. **Energy:**

$$\sum_{i=1}^{N_{g}} \sum_{j=1}^{N_{g}} [\mathrm{M}(\mathrm{i},\mathrm{j})]^{2}$$

1. **Contrast:**

$$\sum_{i=1}^{N_{g}} \sum_{j=1}^{N_{g}} (\mathrm{i}-\mathrm{j})^{2}*M(\mathrm{i},\mathrm{j})$$

1. **Entropy:**

$$\sum_{i=1}^{N_{g}} \sum_{j=1}^{N_{g}} M(\mathrm{i},\mathrm{j})*\log_{2}M(\mathrm{i},\mathrm{j})$$

1. **Homogeneity 1:**

$$\sum_{i=1}^{N_{g}} \sum_{j=1}^{N_{g}} \frac{M(\mathrm{i},\mathrm{j})}{1+|\mathrm{i}-\mathrm{j}|}$$

1. **Homogeneity 2:**

$$\sum_{i=1}^{N_{g}} \sum_{j=1}^{N_{g}} \frac{M(\mathrm{i},\mathrm{j})}{1+|\mathrm{i}-\mathrm{j}|^{2}}$$

1. **Correlation:**

$$\frac{\sum_{i=1}^{N_{g}} \sum_{j=1}^{N_{g}} ijM(\mathrm{i},\mathrm{j})-\mu_{i}(\mathrm{i})\mu_{j}(\mathrm{j})}{\sigma_{x}(\mathrm{i})\sigma_{y}(\mathrm{j})}$$

1. **Variance:**

$$\sum_{i=1}^{N_{g}} \sum_{j=1}^{N_{g}} (\mathrm{i}-\mu)^{2}M(\mathrm{i},\mathrm{j})$$

1. **Sum Average:**

$$\sum_{i=2}^{2N_{g}} iM_{x+y}(\mathrm{i})$$

1. **Sum Entropy:**

$$-\sum_{i=2}^{2N_{g}} M_{x+y}(\mathrm{i})\log_{2}\left[M_{x+y}(\mathrm{i})\right]$$

1. **Dissimilarity:**

$$\sum_{i=1}^{N_{g}} \sum_{j=1}^{N_{g}} |\mathrm{i}-\mathrm{j}|\mathrm{M}(\mathrm{i},\mathrm{j})$$

1. **Inverse Difference Moment:**

$$\sum_{i=1}^{N_{g}} \sum_{j=1}^{N_{g}} \frac{\mathrm{M}(\mathrm{i},\mathrm{j})}{1+(\frac{|\mathrm{i}-\mathrm{j}|^{2}}{N^{2}})}$$

1. **Autocorrelation:**

$$\sum_{i=1}^{N_{g}} \sum_{j=1}^{N_{g}} ijM(\mathrm{i},\mathrm{j})$$

1. **Cluster Prominence**

$$\sum_{i=1}^{N_{g}} \sum_{j=1}^{N_{g}} \left[i+j-u_{x}-u_{y}\right]^{4}M(\mathrm{i},\mathrm{j})$$

1. **Cluster Shade**

$$\sum_{i=1}^{N_{g}} \sum_{j=1}^{N_{g}} \left[i+j-u_{x}-u_{y}\right]^{3}M(\mathrm{i},\mathrm{j})$$

1. **Cluster Tendency**

$$\sum_{i=1}^{N_{g}} \sum_{j=1}^{N_{g}} \left[i+j-u_{x}-u_{y}\right]^{2}M(\mathrm{i},\mathrm{j})$$

1. **Difference Entropy**

$$-\sum_{i=0}^{N_{g}-1} M_{x-y}(i)\log_{2}\left[M_{x-y}(i)\right]$$

1. **Maximum Probability:**

$$\max\left\{M(i,j)\right\}$$

1. **Sum variance**

$$\sum_{i=2}^{2N_{g}} (i-SE)^{2}M_{x+y}(i)$$

1. **Informational measure of correlation 1 (IMC1):**

$$\frac{HXY-HXY1}{\max\left\{HX-HY\right\}}$$

1. **Informational measure of correlation 2 (IMC2):**

$$\sqrt{1-\text{e}^{-2(HXY2-HXY)}}$$

1. **Inverse Difference Moment Normalized (IDMN):**

$$\sum_{i=1}^{N_{g}} \sum_{j=1}^{N_{g}} \frac{M(i,j)}{1+(\frac{\left|i-j\right|^{2}}{N^{2}})}$$

1. **Inverse Difference Normalized (IDN):**

$$\sum_{i=1}^{N_{g}} \sum_{j=1}^{N_{g}} \frac{M(i,j)}{1+(\frac{\left|i-j\right|}{N^{2}})}$$

*Gray Level Run Length Matrix based features (GLRLM)*

GLRLM based features were high-order statistical texture feature, which were defined as *P*(*i, j; θ*) to indicate the number of times j and gray level i appear consecutively in the direction *θ*.

Let:

*P*(*i, j; θ*) be the run-length matrix *P* for a direction *θ*, set *θ=0 and 45*

*N_g_* be the number of discrete intensity values,

*N_r_* be the number of different run lengths, and

*N_p_* be the number of voxels in the ROI.

1. **Short Run Emphasis (SRE):**

$$\frac{\sum_{i=1}^{N_{g}} \sum_{j=1}^{N_{r}} [\frac{P(\mathrm{i},\mathrm{j};\theta)}{j^{2}}]}{\sum_{i=1}^{N_{g}} \sum_{j=1}^{N_{r}} P(\mathrm{i},\mathrm{j};\theta)}$$

1. **Long Run Emphasis (LRE):**

$$\frac{\sum_{i=1}^{N_{g}} \sum_{j=1}^{N_{r}} j^{2}P(\mathrm{i},\mathrm{j};\theta)}{\sum_{i=1}^{N_{g}} \sum_{j=1}^{N_{r}} P(\mathrm{i},\mathrm{j};\theta)}$$

1. **Gray-Level Nonuniformity (GLN):**

$$\frac{\sum_{i=1}^{N_{g}} [\sum_{j=1}^{N_{r}} P(\mathrm{i},\mathrm{j};\theta)]^{2}}{\sum_{i=1}^{N_{g}} \sum_{j=1}^{N_{r}} P(\mathrm{i},\mathrm{j};\theta)}$$

1. **Run-Length Nonuniformity (RLN):**

$$\frac{\sum_{j=1}^{N_{r}} [\sum_{i=1}^{N_{g}} P(\mathrm{i},\mathrm{j};\theta)]^{2}}{\sum_{i=1}^{N_{g}} \sum_{j=1}^{N_{r}} P(\mathrm{i},\mathrm{j};\theta)}$$

1. **Run Percentage (RP):**

$$\sum_{i=1}^{N_{g}} \sum_{j=1}^{N_{r}} \frac{P(\mathrm{i},\mathrm{j};\theta)}{N_{p}}$$

1. **Low Gray-Level Run Emphasis (LGRE):**

$$\frac{\sum_{i=1}^{N_{g}} \sum_{j=1}^{N_{r}} [\frac{P(\mathrm{i},\mathrm{j};\theta)}{i^{2}}]}{\sum_{i=1}^{N_{g}} \sum_{j=1}^{N_{r}} P(\mathrm{i},\mathrm{j};\theta)}$$

1. **High Gray-Level Run Emphasis (HGRE):**

$$\frac{\sum_{i=1}^{N_{g}} \sum_{j=1}^{N_{r}} i^{2}P(\mathrm{i},\mathrm{j};\theta)}{\sum_{i=1}^{N_{g}} \sum_{j=1}^{N_{r}} P(\mathrm{i},\mathrm{j};\theta)}$$

1. **Short Run Low Gray-Level Emphasis (SRLGE):**

$$\frac{\sum_{i=1}^{N_{g}} \sum_{j=1}^{N_{r}} \frac{P(\mathrm{i},\mathrm{j};\theta)}{i^{2}j^{2}}}{\sum_{i=1}^{N_{g}} \sum_{j=1}^{N_{r}} P(\mathrm{i},\mathrm{j};\theta)}$$

1. **Short Run High Gray-Level Emphasis (SRHGE):**

$$\frac{\sum_{i=1}^{N_{g}} \sum_{j=1}^{N_{r}} \frac{i^{2}P(\mathrm{i},\mathrm{j};\theta)}{j^{2}}}{\sum_{i=1}^{N_{g}} \sum_{j=1}^{N_{r}} P(\mathrm{i},\mathrm{j};\theta)}$$

1. **Long Run Low Gray-Level Emphasis (LRLGE):**

$$\frac{\sum_{i=1}^{N_{g}} \sum_{j=1}^{N_{r}} \frac{j^{2}P(\mathrm{i},\mathrm{j};\theta)}{i^{2}}}{\sum_{i=1}^{N_{g}} \sum_{j=1}^{N_{r}} P(\mathrm{i},\mathrm{j};\theta)}$$

1. **Long Run High Gray-Level Emphasis (LRHGE):**

$$\frac{\sum_{i=1}^{N_{g}} \sum_{j=1}^{N_{r}} i^{2}j^{2}P(\mathrm{i},\mathrm{j};\theta)}{\sum_{i=1}^{N_{g}} \sum_{j=1}^{N_{r}} P(\mathrm{i},\mathrm{j};\theta)}$$

1. **Mean:**

$$\frac{1}{2N_{\text{g}}}\sum_{i=1}^{N_{g}} \sum_{j=1}^{N_{g}} [P(\mathrm{i},\mathrm{j})]^{2}$$

1. **Entropy:**

$$\sum_{i=1}^{N_{g}} \sum_{j=1}^{N_{g}} P(\mathrm{i},\mathrm{j})*\log_{2}P(\mathrm{i},\mathrm{j})$$

1. **Energy:**

$$\sum_{i=1}^{N_{g}} \sum_{j=1}^{N_{g}} [P(\mathrm{i},\mathrm{j})]^{2}$$

*Gray Level Size Zone Matrix based features (GLSZM)*

GLSZM based features were high-order statistical texture features, which were defined as *P*(*i, j*) to indicate the areas of size j and gray level i.

Let:

*P*(*i, j*) be the size zone of matrix *P*,

*N_g_* be the number of discrete intensity values,

*N_r_* be the number of different areas sizes,

*N_p_* be the number of voxels in the ROI.

1. **Small Zone Emphasis (SZE):**

$$\frac{\sum_{i=1}^{N_{g}} \sum_{j=1}^{N_{r}} [\frac{P(\mathrm{i},\mathrm{j})}{j^{2}}]}{\sum_{i=1}^{N_{g}} \sum_{j=1}^{N_{r}} P(\mathrm{i},\mathrm{j})}$$

1. **Large Zone Emphasis (LZE):**

$$\frac{\sum_{i=1}^{N_{g}} \sum_{j=1}^{N_{r}} j^{2}P(\mathrm{i},\mathrm{j})}{\sum_{i=1}^{N_{g}} \sum_{j=1}^{N_{r}} P(\mathrm{i},\mathrm{j})}$$

1. **Gray-Level Nonuniformity (GLN):**

$$\frac{\sum_{i=1}^{N_{g}} [\sum_{j=1}^{N_{r}} P(\mathrm{i},\mathrm{j})]^{2}}{\sum_{i=1}^{N_{g}} \sum_{j=1}^{N_{r}} P(\mathrm{i},\mathrm{j})}$$

1. **Zone-Size Nonuniformity (ZSN):**

$$\frac{\sum_{j=1}^{N_{r}} [\sum_{i=1}^{N_{g}} P(\mathrm{i},\mathrm{j})]^{2}}{\sum_{i=1}^{N_{g}} \sum_{j=1}^{N_{r}} P(\mathrm{i},\mathrm{j})}$$

1. **Zone Percentage (ZP):**

$$\sum_{i=1}^{N_{g}} \sum_{j=1}^{N_{r}} \frac{P(\mathrm{i},\mathrm{j})}{N_{p}}$$

1. **Low Gray-Level Zone Emphasis (LGZE):**

$$\frac{\sum_{i=1}^{N_{g}} \sum_{j=1}^{N_{r}} [\frac{P(\mathrm{i},\mathrm{j})}{i^{2}}]}{\sum_{i=1}^{N_{g}} \sum_{j=1}^{N_{r}} P(\mathrm{i},\mathrm{j})}$$

1. **High Gray-Level Zone Emphasis (HGZE):**

$$\frac{\sum_{i=1}^{N_{g}} \sum_{j=1}^{N_{r}} i^{2}P(\mathrm{i},\mathrm{j})}{\sum_{i=1}^{N_{g}} \sum_{j=1}^{N_{r}} P(\mathrm{i},\mathrm{j})}$$

1. **Small Zone Low Gray-Level Emphasis (SZLGE):**

$$\frac{\sum_{i=1}^{N_{g}} \sum_{j=1}^{N_{r}} \frac{P(\mathrm{i},\mathrm{j})}{i^{2}j^{2}}}{\sum_{i=1}^{N_{g}} \sum_{j=1}^{N_{r}} P(\mathrm{i},\mathrm{j})}$$

1. **Small Zone High Gray-Level Emphasis (SZHGE):**

$$\frac{\sum_{i=1}^{N_{g}} \sum_{j=1}^{N_{r}} \frac{i^{2}P(\mathrm{i},\mathrm{j})}{j^{2}}}{\sum_{i=1}^{N_{g}} \sum_{j=1}^{N_{r}} P(\mathrm{i},\mathrm{j})}$$

1. **Large Zone Low Gray-Level Emphasis (LZLGE):**

$$\frac{\sum_{i=1}^{N_{g}} \sum_{j=1}^{N_{r}} \frac{j^{2}P(\mathrm{i},\mathrm{j})}{i^{2}}}{\sum_{i=1}^{N_{g}} \sum_{j=1}^{N_{r}} P(\mathrm{i},\mathrm{j})}$$

1. **Large Zone High Gray-Level Emphasis (LZHGE):**

$$\frac{\sum_{i=1}^{N_{g}} \sum_{j=1}^{N_{r}} i^{2}j^{2}P(\mathrm{i},\mathrm{j})}{\sum_{i=1}^{N_{g}} \sum_{j=1}^{N_{r}} P(\mathrm{i},\mathrm{j})}$$

1. **Gray-Level Variance (GLV):**

$$\sum_{i=1}^{N_{g}} \sum_{j=1}^{N_{r}} \{\mathrm{iP}(\mathrm{i},\mathrm{j})-\frac{\sum_{i=1}^{N_{g}} i[\sum_{j=1}^{N_{r}} P(\mathrm{i},\mathrm{j})]}{N_{g}N_{r}}\}$$

1. **Zone-Size Variance (ZSV):**

$$\sum_{i=1}^{N_{g}} \sum_{j=1}^{N_{r}} \{\mathrm{jP}(\mathrm{i},\mathrm{j})-\frac{\sum_{i=1}^{N_{g}} i[\sum_{j=1}^{N_{r}} P(\mathrm{i},\mathrm{j})]}{N_{g}N_{r}}\}$$

*Neighborhood Gray Tone Difference Matrix based features (NGTDM)*

NGTDM based features were high-order statistical texture features, which were defined as *S(i)* to indicate the sum of the absolute value between gray intensity level i and it’s neighbors’ average intensity.

Let:

*S(i)* be the sum of absolute value between gray intensity level i and its neighbors’ average intensity,

*C(i)* be the number of voxels with the gray intensity level I,

*N_g_* be the number of discrete intensity values.

1. **Coarseness:**

$$\frac{1}{\varepsilon+\sum_{i=1}^{N_{g}} \frac{C(\mathrm{i})\mathrm{S}(\mathrm{i})}{\sum_{i=1}^{N_{g}} C(\mathrm{i})}}$$

1. **Contrast:**

$$\frac{\sum_{i=1}^{N_{g}} \sum_{j=1}^{N_{g}} C(\mathrm{i})\mathrm{C}(\mathrm{j})(\mathrm{i}-\mathrm{j})^{2}}{(\sum_{i=1}^{N_{g}} C(\mathrm{i}))^{2}}*\sum_{i=1}^{N_{g}} S(\mathrm{i})*\frac{1}{N_{g}(\mathrm{N}_{g}-1)\sum_{i=1}^{N_{g}} C(\mathrm{i})}$$

1. **Busyness:**

$$\frac{\sum_{i=1}^{N_{g}} C(\mathrm{i})\mathrm{S}(\mathrm{i})}{\sum_{i=1}^{N_{g}} \sum_{j=1}^{N_{g}} (\mathrm{iC}(\mathrm{i})-\mathrm{jC}(\mathrm{j}))}, C(\mathrm{i})\neq0,\mathrm{C}(\mathrm{j})\neq0$$

1. **Complexity:**

$$\frac{1}{(\sum_{i=1}^{N_{g}} C(\mathrm{i}))^{2}}*\sum_{i=1}^{N_{g}} \sum_{j=1}^{N_{g}} \frac{|\mathrm{i}-\mathrm{j}|(\mathrm{C}(\mathrm{i})\mathrm{S}(\mathrm{i})+\mathrm{C}(\mathrm{j})\mathrm{S}(\mathrm{j}))}{(\mathrm{C}(\mathrm{i})+\mathrm{C}(\mathrm{j}))}, C(\mathrm{i})\neq0,\mathrm{C}(\mathrm{j})\neq0$$

1. **Strength:**

$$\frac{\sum_{i=1}^{N_{g}} \sum_{j=1}^{N_{g}} (\mathrm{C}(\mathrm{i})-\mathrm{C}(\mathrm{j}))*(\mathrm{i}-\mathrm{j})^{2}}{\sum_{i=1}^{N_{g}} C(\mathrm{i})*\sum_{i=1}^{N_{g}} S(\mathrm{i})}, C(\mathrm{i})\neq0,\mathrm{C}(\mathrm{j})\neq0$$

**(4) Wavelet features: first order statistical and texture features of a wavelet filtered image.**

A total of 4535 wavelet based features were extracted for each sequence.

With the Gabor wavelet, we obtained five different wave lengths and eight different orientations filtered images. These wavelet-based features were computed on the filtered images. The original image was filtered by a two-dimensional Gabor filter defined as:

$$\begin{matrix} \text{g}(x,y,\lambda,\theta,\psi,\sigma,\gamma)=e^{-\frac{x'^{2}+\gamma^{2}y'^{2}}{2\sigma^{2}}}e^{i(2\pi\frac{x'}{\lambda}+\psi)} \\ x'=x\cos\theta+y\sin\theta\\ y'=-x\sin\theta+y\cos\theta\\ \psi=2.5*\pi/2 \\ \sigma=\sqrt{2} \\ \gamma=1.5*\pi\end{matrix}$$

Here, five wave lengths $\lambda=0,1,2,3,4$ and eight orientations$\theta=\frac{j-1}{8}\pi, j=1,2,....,8$ were used. After filtering, 40 filtered images were generated. For each image, the first order statistical and texture features were computed. Finally, 4280 Gabor wavelet based features were extracted.

With the Law’s filter, we obtained fifteen filtered images. The filtered images were defined as follows:

L5L5,E5L5,E5E5,S5L5,S5E5,S5S5,W5L5,W5E5,W5S5,W5W5,R5L5,R5S5,R5W5,R5R5

Here, it meant as follows:

$$\left\{\begin{matrix} L5=\left[\frac{1}{16},\frac{4}{16},\frac{6}{16},\frac{4}{16},\frac{1}{16}\right] \\ E5=\left[-1,-2,0,2,1\right] \\ S5=\left[-1,0,2,0,-1\right] \\ W5=\left[-1,2,0,-2,1\right] \\ R5=\left[1,-4,6,-4,1\right] \end{matrix}\right.\to eg.R5R5\left[\begin{matrix} -1 & -4 & -6 & -4 & -1 \\ -2 & -8 & -12 & -8 & -2 \\ 0 & 0 & 0 & 0 & 0 \\ 2 & 8 & 12 & 8 & 2 \\ 1 & 4 & 6 & 4 & 1 \end{matrix}\right]$$

After filtering, 15 filtered images were generated. For each image, the 17 first order statistic features were computed. Finally, 255 Law’s wavelet based features were extracted.

**A2. Supplementary Tables**

**Table S1. The relationship between the selected radiomics features and the pathological types.**

| Name | R-volume | P-volume |
| --- | --- | --- |
| gabor3_glszm_SZHGE | 0.045 | 0.613 |
| gabor7_glcm_cluster_shade | 0.006 | 0.947 |
| gabor14_glcm_cluster_tendency | 0.109 | 0.216 |
| gabor18_glcm_IMC2 | 0.052 | 0.557 |
| gabor18_glszm_LZLGE | 0.054 | 0.544 |
| gabor29_glszm_SZSE | -0.022 | 0.802 |
| gabor29_glszm_SZHGE | -0.039 | 0.661 |
| gabor30_glszm_SZHGE | -0.010 | 0.908 |
| gabor35_glszm_SZLGE | 0.090 | 0.307 |
| gabor36_glrlm_LGLRE | 0.005 | 0.952 |
| gabor36_glrlm45_LGLRE | 0.003 | 0.971 |
| gabor36_glszm_LGLZE | 0.045 | 0.608 |
| W5S5_fos_skewness | -0.026 | 0.767 |
| W5S5_fos_mass | -0.088 | 0.318 |
| W5W5_fos_mean | -0.170 | 0.053 |
| W5W5_fos_mass | -0.159 | 0.070 |
| R5S5_fos_median | 0.156 | 0.076 |

**Table S2. The results of T-tests between the radiomics features and the brain regions.**

| Name | Frontal lobe | Temporal lobe | Parietal lobe | Insula |
| --- | --- | --- | --- | --- |
| gabor3_glszm_SZHGE | 0.022* | 0.453 | 0.452 | 0.389 |
| gabor7_glcm_cluster_shade | 0.793 | 0.027* | 0.374 | 0.025* |
| gabor14_glcm_cluster_tendency | 0.735 | 0.700 | 0.889 | 0.003* |
| gabor18_glcm_IMC2 | 0.303 | 0.049* | 0.557 | 0.014* |
| gabor18_glszm_LZLGE | 0.987 | 0.436 | 0.582 | 0.743 |
| gabor29_glszm_SZSE | 0.068 | 0.024* | 0.483 | 0.859 |
| gabor29_glszm_SZHGE | 0.106 | 0.366 | 0.210 | 0.365 |
| gabor30_glszm_SZHGE | 0.877 | 0.223 | 0.051 | 0.299 |
| gabor35_glszm_SZLGE | 0.150 | 0.995 | 0.399 | 0.555 |
| gabor36_glrlm_LGLRE | 0.338 | 0.011* | 0.986 | 0.107 |
| gabor36_glrlm45_LGLRE | 0.223 | 0.004* | 0.960 | 0.058 |
| gabor36_glszm_LGLZE | 0.604 | 0.522 | 0.660 | 0.768 |
| W5S5_fos_skewness | 0.277 | 0.597 | 0.023* | 0.432 |
| W5S5_fos_mass | 0.633 | 0.162 | 0.395 | 0.864 |
| W5W5_fos_mean | 0.537 | 0.590 | 0.912 | 0.432 |
| W5W5_fos_mass | 0.764 | 0.411 | 0.771 | 0.472 |
| R5S5_fos_median | 0.499 | 0.636 | 0.305 | 0.300 |

Note, the asterisk means that this radiomics feature was different significantly between the certain brain region when was involved in or not.

**Table S3. The relationship between each radiomics feature and the tumor volume.**

| Name | R-volume | P-volume |
| --- | --- | --- |
| gabor3_glszm_SZHGE | -0.437 | <0.001* |
| gabor7_glcm_cluster_shade | -0.401 | <0.001* |
| gabor14_glcm_cluster_tendency | -0.093 | 0.294 |
| gabor18_glcm_IMC2 | -0.458 | <0.001* |
| gabor18_glszm_LZLGE | 0.564 | <0.001* |
| gabor29_glszm_SZSE | -0.178 | 0.043 |
| gabor29_glszm_SZHGE | -0.317 | <0.001* |
| gabor30_glszm_SZHGE | -0.383 | <0.001* |
| gabor35_glszm_SZLGE | -0.249 | 0.004 |
| gabor36_glrlm_LGLRE | -0.179 | 0.042 |
| gabor36_glrlm45_LGLRE | -0.200 | 0.022 |
| gabor36_glszm_LGLZE | -0.078 | 0.379 |
| W5S5_fos_skewness | 0.185 | 0.035 |
| W5S5_fos_mass | 0.187 | 0.033 |
| W5W5_fos_mean | 0.067 | 0.447 |
| W5W5_fos_mass | 0.059 | 0.505 |
| R5S5_fos_median | -0.101 | 0.251 |

Note, the asterisk means that this radiomics feature was still significant after multiple correlation.

Table S4. The study details according to IBSI reporting guidelines.

| Topic | Item | Description |
| --- | --- | --- |
| **Patient** |  |  |
| Region of interest | 1 | Low grade gliomas tumor area. |
| **Acquisition** |  |  |
| Acquisition protocol | 6 | A standard imaging protocol was used. |
| Scanner type | 7 | Siemens |
| Imaging modality | 8 | MRI |
| Static/dynamic scans | 9a | Static |
| RF coil | 17 | A 12-channel receive-only head coil. |
| Scanning sequence | 18a | T2-weight MRI. |
| Repetition time | 19 | 5800 ms |
| Echo time | 20 | 110 ms |
| Flip angle | 23 | 150 degrees |
| Acquisition type | 24 | 3D |
| Magnetic field strength | 27 | 3.0T |
| **Reconstruction** |  |  |
| In-plane resolution | 28 | Field of view = 240$\times$188 mm^2^, matrix = 384$\times$300 |
| Image slice thickness | 29 | 5.0 mm |
| **Segmentation** |  |  |
| Segmentation method | 48a | Semi-automatically segmented |
|  | 48b | Two neuroradiologists segmented the ROI, and two other board-certified experts reviewed the segmentations using imaging features in combination with seizure history, clinical examination, neuroimaging data to solve any discrepancies. The areas with abnormal hyperintense signals on the images were identified as tumor volumes, and the cerebrospinal fluid signals should not be involved in. |
|  | 48c | Tumors were semi-automatically segmented using the ITK-SNAP software. |
| **Image processing -discretization** |  |  |
| Discretisation method | 56a | Fixed bin number |
|  | 56b | 25 |
| **Image processing** **- image transformation** |  |  |
| Image filter | 57 | 2D Gabor filter |
| **Image biomarker computation** |  |  |
| Biomarker set | 58 |  |
| IBSI compliance | 59 | The software used to extract the set of image biomarkers is able to reproduce the IBSI feature reference values. |
| Robustness | 60 |  |
| Software availability | 61 | An in-house software written in MATLAB 2017b (MathWorks, Inc., Natick, MA, USA). |
| **Image biomarker computation – texture parameters** |  |  |
| Texture matrix aggregation | 62 | BTW3 and 8QNN |
| Distance weighting | 63 | No weighting |
| CM symmetry | 64 | Symmetric |
| CM distance | 65 | δ = 1 |
| SZM linkage distance | 66 | Chebyshev distance of 1 |
| NGTDM distance | 69 | Chebyshev distance of 1 |
| **Machine learning and radiomics analysis** |  |  |
| Diagnostic and pro-  gnostic modelling | 72 | Prognostic modeling. |
| Comparison with known  factors | 73 | In the method and result sections. |
| Multicollinearity | 74 | In the feature selection section. |
